# Precision therapy for acute myeloid leukemia

**DOI:** 10.1186/s13045-017-0543-7

**Published:** 2018-01-05

**Authors:** Xue Yang, Jianxiang Wang

**Affiliations:** State Key Laboratory of Experimental Hematology, Institute of Hematology and Blood Diseases Hospital, Chinese Academy of Medical Sciences and Peking Union Medical College, Tianjin, China

**Keywords:** Acute myeloid leukemia, Precision therapy, Conventional chemotherapies, Molecular targeted inhibitors, Epigenetic mutations, Immunotherapy

## Abstract

Acute myeloid leukemia (AML) is a molecularly and clinically heterogeneous disease. Despite advances in understanding the pathogenesis of AML, the standard therapy remained nearly unchanged over the past three decades. With the poor survival for older patients and high relapse rate, multiple studies are ongoing to address this important issue. Novel therapies for AML, including the refinements of conventional cytotoxic chemotherapies and genetic and epigenetic targeted drugs, as well as immunotherapies, have been developed in recent years. Here, we present a mechanism-based review of some promising new drugs with clinical efficacy, focus on targeted drugs that are most potential to pave the road to success, and put forward the major challenges in promoting the precision therapy for AML.

## Background

Acute myeloid leukemia (AML) represents a heterogeneous malignancy characterized by a clonal proliferation and impaired differentiation of myeloid precursors with diverse outcomes. Despite the advances in understanding the molecular heterogeneity and pathogenesis of AML, there has been little progress in the standard therapy for AML over the past four decades. The classic treatment ranges from cytarabine-based chemotherapy to hematopoietic stem cell transplantation (HSCT), with a 5-year overall survival (OS) of 40% for patients younger than 60 years. For those older than 60 years, who made up of the majority of AML cases, the 5-year OS was only 10~20% [1, 2]. Few of patients who relapsed after complete remission (CR) could survive for more than 5 years [3].

Briefly, the treatment of AML consists mainly of remission induction and post-remission therapy which contains chemotherapy, targeted therapies, and HSCT. In terms of induction therapy, for adult patients with newly diagnosed AML, a combination of anthracycline for 3 days and standard-dose cytarabine for 7 to 10 days (“7 + 3” therapy) are recommended. For elder patients (> 60 years), the best chemotherapy remains to be identified. Most of them recommended the same remission induction regimen except those with unfavorable risk or severe commodity who are too fragile to tolerate intensive chemotherapy. When it comes to post-remission treatment including consolidation and maintenance therapy, risk stratification should be taken into consideration. AML patients are categorized based on cytogenetic, molecular, and clinical characteristics that are prognostic important. For younger patients, high-dose cytarabine are recommended in patients with favorable cytogenesis. While for those with adverse prognosis, allogeneic HSCT (allo-HSCT) should be performed in the first remission. For elder patients who fit for chemotherapy in the first complete remission (CR), consolidation therapy could contain anthracycline and cytarabine or intermediate-dose cytarabine alone. Likewise, those with unfavorable risk should be considered for nonmyeloablative HCT. Of note, for relapsed or refractory (R/R) AML population, allo-HSCT provides the highest likelihood of cure.

Among all AML subtypes, acute promyelocytic leukemia (APL) contributes the highest proportion of cure rate for patients undergoing targeted therapy such as all-transretinoic acid (ATRA) and arsenic trioxide, which implies a strong need for individualized medicine. With the advent in next-generation sequencing technologies, novel therapies have emerged, including multiple molecular target inhibitors and immunotherapies [Table 1].

**Table 1:** Examples of targeted drugs for AML

Target	Drug	Phase of development
PLKs	Volasertib	3
FLT3	Sorafenib	2
	Midostaurin (PKC412)	3
	Quizartinib (AC220)	3
	Crenolanib (CP868596)	2
	Gilteritinib (ASP2215)	3
	Lestaurtinib (CEP-701)	3
DNMTs	Azacitidine (5-Aza)	Approved
	Decitabine	Approved
	Guadecitabine (SGI-110)	3
	Sapacitabine (CYC682)	3
IDH2	AG-221	3
IDH1	AG-120	2
HDACs	Vorinostat	3
	Entinostat	2
BET	OTX015	1
DOT1L	Pinometostat (EPZ-2676)	1
LSD1	GSK2879552	1
CD33	GO	3
	SGN-33A	3
	CD33 CART	Preclinical
CD123	CSL362	Preclinical
	SL-401	Preclinical
	CD123 CART	Preclinical
PD-1	Nivolumab	2
CTLA4	Ipilimumab	2

*PLKs* polo-like kinases, *FLT3* Fms-like tyrosine kinase 3, *DNMTs* DNA methyltransferases, *IDH* isocitrate dehydrogenase, *HDACs* histone deacetylases, *BET* bromodomain and extra-terminal motif, *DOT1L* disruptor of telomeric silencing 1-like, *LSD1* lysine-specific histone demethylase 1A, *PD-1* programmed cell death protein 1, *CTLA4* cytotoxic T-lymphocyte-associated protein 4

## Heterogeneity of AML

Tumor heterogeneity refers to distinct morphological and phenotypic features in tumors mainly arising from gene alterations, which has been observed in all types of tumors including leukemia. The heterogeneity of AML involves both genomic and epigenomic changes, including distinct sets of cytogenetic abnormalities and somatic mutations [4], resulting in a range of morphological, immunophenotypic, cytogenetic, biomolecular, and clinical features [5]. Moreover, in the course of the disease, the leukemic clone may change from diagnosis to relapse due to the heterogeneity of leukemia cells [6], and transformation of hematopoietic stem cells (HSCs) to leukemia initiating cells occurs at different stages within primitive multipotent cells [7, 8]. Consequently, the heterogeneity of AML brings about varied response to treatment, as well as drug resistance and disease relapse, posing a challenge to personalized therapeutic regimens, likewise, known as precision medicine.

## Refinements of conventional cytotoxic chemotherapies

Conventional intensive chemotherapy has a cure rate of only 30–50%, and the majority of patients aged 70 years or older could not benefit from it due to poor tolerance and high mortality [9]. Despite 40–80% patients achieving CR, the median survival in elderly patients receiving intensive chemotherapy is 4.6 months with a 1-year survival rate of only 28% [10]. Besides, a high frequency of subsequent relapse remains the major obstacle to overcome. In the past decades, studies have driven the improvements in OS by novel formulations and refinements of conventional chemotherapy.

### Intensification of the standard induction therapy

The 7 + 3 regimen, consisting of 7 days continuous infusion of cytarabine along with a short infusion or bolus of an anthracycline given on days 1 through 3, has been known as standard induction therapy in AML in the past decades. Recently, the escalation of daunorubicin or cytarabine dose have shown benefit in the induction therapy. In adults under 60 years of age, previous trials have suggested that a daunorubicin dose of 90 mg is superior to 45 mg, with the former showing an improved remission rate and survival benefit [11]. In older patients (> 60 years), a Korea trial showed a significant benefit of 90 mg/m^2^ both in remission rate and OS [12], which was particularly prominent in intermediate-risk patients in the ECOG1900 trial. In a randomized AML17 trail comparing 90 mg/m^2^ with 60 mg/m^2^, no significant difference was seen in remission rate or OS in any cytogenetic subgroup, with the 60-day mortality rate significantly higher in the high-dose (HD) daunorubicin group (90 mg/m^2^) (10 vs 5%, *P* = 0.001). However, it still remains necessary to take notice of molecular subgroups when it comes to longer follow-up, since in a recent E1900 trial with a median follow-up of 80 months, patients with Fms-like tyrosine kinase 3 (FLT3), nucleophosmin (NPM1), and DNA methyltransferase (DNMT)3A all benefited from HD daunorubicin [13].

In addition to daunorubicin, the administration of cytarabine at a daily dose of 100 to 200 mg/m^2^ for 7 to 10 days is also an important part in the standard induction therapy. In the EORTC-GIMEMA AML-12 trial with a median follow-up of 6 years in patients aged 15 to 60 years, higher remission and survival rate were observed in high-dose cytarabine (3000 mg/m^2^ per 12 h on days 1, 3, 5, and 7) without significant toxicity. Particularly, for patients under 46 years old, the CR rate, event-free survival (EFS), and OS in high-dose cytarabine arm were significantly higher than standard dose cytarabine arm (100 mg/m^2^ per day continuously for 10 days) [14].

An alternative method to intensify standard induction regimen is the addition of a purine analog such as fludarabine or cladribine. One study demonstrated that the DAC regimen (DA plus cladribine), rather than DAF regimen (DA plus fludarabine), is associated with improved OS and CR in patients younger than 60 years with newly diagnosed AML. It is worth mentioning that both DAF and DAC increased CR rate in AML patients with adverse karyotype. Thus, further trials focusing on particular subgroups are needed [15]. Clofarabine is a second-generation purine analog, which has shown effectiveness in both R/R AML patients and newly diagnosed older patients [16, 17]. A phase III study in newly diagnosed AML patients aged 18 to 65 years old confirmed the potent efficacy of clofarabine integrated in standard induction treatment, which showed reduced relapse probability without survival improvement. The study only found the survival benefit of clofarabine in subgroups of intermediate-risk AML and AML genotype without NPM1 and FLT3-ITD mutations [18].

### CPX-351

CPX-351 is designed as a liposomal formulation of 7 + 3 combination in a 5:1 ratio of cytarabine and daunorubicin, which was proved to be an optimal combination, with the highest level of synergy and the lowest level of antagonism [19, 20]. Two phase II randomized studies in 127 and 125 patients both confirmed a higher rate of CR (66.7 vs 51.2%, and 49.3 vs 40.9%, respectively) for patients treated with CPX-315 compared with those receiving 7 + 3 regimen, and no difference in EFS or OS has been found in both phase II trials [21, 22]. However, it is worth mentioning that a phase III study, in which the studying group was not strictly limited, demonstrated an increased OS with daunorubicin in AML patients between the age of 60 and 65 years [23]. Another phase III study to confirm the efficacy of CPX-351 as first-line therapy in elderly patients (60–75 years) with high-risk (secondary) AML is ongoing (NCT01696084), which may make CPX-351 a better induction therapy for elderly patients who are not suitable for chemotherapy.

### Vosaroxin

Vosaroxin is a quinolone derivative that intercalates DNA and inhibits topoisomerase II without producing oxygen free radicals, which has been confirmed to have better efficacy and lower cardiac toxicity than traditional anthracyclines [24]. Most recently, a large multicenter randomized phase III trial with 711 patients named VALOR demonstrated that the addition of vosaroxin to cytarabine resulted in a significant improvement in CR (30.1 vs 16.3%, *P* < 0.0001) and OS (6.7 vs 5.3 months, *P* = 0.024) when censored for HSCT in R/R AML patients ≥ 60 years [25]. With favorable efficacy and tolerability among older patients as well as notable survival benefits in subsets, vosaroxin stands a nice choice for novel combinatorial regimens, which will be further confirmed by future trials.

## Molecular targeted inhibitors

### Volasertib

Volasertib (also known as BI 6727), which was awarded orphan drug status for AML in 2014, is a small-molecular inhibitor of polo-like kinases (PLKs), particularly PLK-1 (which was listed on Table 1). Inhibition of PLK1 overexpression in AML cell lines can bring about disorganized centrosome maturation, spindle assembly and cytokinesis during mitosis [20], and then cellular apoptosis subsequently. A phase II study made a comparison between the combination of volasertib with low-dose cytarabine (LDAC) and LDAC alone. The result confirmed greater clinical efficacy in the combination arm, statistically significant in CR (30 vs 13.3%, *P* = 0.052), median EFS (5.6 vs 2.3 months, *P* = 0.021), and median OS (8 vs 5.2 months, *P* = 0.047) [26]. Meanwhile, there is also an ongoing phase III trial (NCT01721876) and a phase II trial of intensive chemotherapy with or without volasertib (NCT02198482).

### FLT3 inhibitors

FLT3 is a class III tyrosine kinase receptor that stimulates normal hematopoiesis and cell proliferation in primitive hematopoietic stem and progenitor cells [27]. Although activating mutations in FLT3 are reported in only 30% of AML adults [28], FLT3 is constitutively expressed by autocrine signaling on leukemic cells in 70–100% of AML patients [29]. There are two types of FLT3 mutations, including approximately 20% of internal tandem duplications (FLT3/ITD) and 5~10% of point mutations in activating loop of tyrosine kinase domain (FLT3/TKD), constitutively activating cell proliferation and survival of leukemia blasts. Both mutations are associated with poor prognosis and outcome, particularly FLT3/ITD, with an estimation of 2-year disease-free survival (DFS) rates of 20% and 4-year OS of 20% [30]. Of note, ITD mutations are associated with a poor prognosis due to a high relapse rate, and higher allelic ratios of mutated/wild-type variants confer a worse prognosis [31], suggesting a greater clinical response to selective FLT3-inhibitors [32]. Recent years have witnessed a growing development of several FLT3 inhibitors tested in clinical trials as either single agent or in combination with conventional chemotherapies, with the former usually associated with modest anti-tumor activity, transient reduction of blasts, and increased toxicity [33]. Though more tolerated than traditional cytotoxic agents, drug resistance has still posed a major challenge to patients treated with single FLT3 inhibitor, including F691, N676, and D835 mutation with kinase domain of FLT3-ITD [34].

#### Sorafenib

Sorafenib is a potent first-generation multikinase inhibitor with activity against FLT3/ITD receptor, which has been evaluated as either single agent [35–41] or in combination with chemotherapies [42–45]. SORAML is a placebo-controlled randomized study of adding sorafenib to daunorubicin and cytarabine (7 + 3) in 267 newly diagnosed patients aged 18–60 years. The addition of sorafenib resulted in a significantly prolonged 3-year EFS (40 vs 22%, *P* = 0.013) and RFS (56 vs 38%, *P* = 0.017) without improvement in OS and CR [46]. In contrast to this study, a second randomized study in 201 older patients aged 61–80 years showed no improvement in EFS, CR, and OS, with a higher early mortality (17 versus 7%, *P* = 0.052) compared with placebo [44]. It can be seen from the difference of two studies that the combination of sorafenib with intensive chemotherapy may be too toxic for older patients, who have a poor prognosis mainly due to more resistance and less tolerance. Thus, for older patients, combining multikinase inhibitors with lower intensity therapies like hypomethylating agents (HMAs) may be an alternative choice [47]. Recent studies also suggest potential benefit of post-HSCT sorafenib in patients with FLT3-ITD [48].

#### Midostaurin (PKC412)

Midostaurin is a first-generation multi-target agent that inhibits FLT3, c-kit, platelet-derived growth factor receptor (PDGFR), vascular endothelial growth factor receptor (VEGFR), and protein kinase C [3]. As a well-tolerated and orally bioavailable agent, it enhances the response to induction chemotherapy and represents the potential to bridge mutant and wild-type (WT)-FLT3 AML patients to transplantation [49]. In two phase IIB studies of single-agent midostaurin administered in FLT3-mutated and FLT3-WT AML patients, there is a blast decrease ≥ 50% in the majority of R/R or vulnerable/frail patients, especially those with FLT3 mutation, but CRs are rare and transient [50, 51]. When it comes to combination, a phase IB trail adding midostaurin of two doses during (concomitant) or after (sequential) standard induction therapy confirmed a higher CR and lower toxicity in the lower-dose group (50 mg daily), as well as a higher CR rate in FLT3-mutated patients (92 vs 74%) [52]. In the meantime, there was a multicenter, randomized phase III trial (RATIFY) in 717 younger adult patients, which demonstrated a significant improvement in OS and EFS among AML patients with FLT3 mutation, when adding midostaurin to standard induction therapy. In particular, the benefit of midostaurin was observed in patients undergoing transplantation during the first remission [53]. Thus, the combination regimen could be considered as first-line treatment in younger AML patient, while it is still uncertain whether the combination regimen might benefit older patients or those with wild-type FLT3. As is reported, combination with histone deacetylase (HDAC) inhibitors is also associated with a higher CR [54–56], and most recently, whether midostaurin improves RFS after transplant is under investigation (NCT01883362).

#### Quizartinib (AC220)

Quizartinib selectively inhibits FLT3/STK1, CSF1R/FMS, SCFR/KIT, and PDGFRs. A phase I trial in R/R AML patients determined the maximum tolerated dose (MTD) of 200 mg per day with the dose-limiting toxicity (DLT) of grade 3 QTc prolongation [57]. Subsequently, several phase II trials studying with lower doses demonstrated prominent composite CR (CRc) rate ranging from 44 to 54% and ORR (CRc + PR) ranging from 61 to 72% in FLT3-ITD-positive patients [58–60]. Combination studies are ongoing (NCT01892371). As mentioned, single agent is proved to have limited efficacy due to drug resistance. Though active against FLT3-ITD mutation, most of tyrosine kinase inhibitors (TKIs) including quizartinib had no activity against FLT3-TKD mutation [34], the effect of which on the outcome remains unsettled.

#### Crenolanib (CP868596)

To maximize tolerability and response duration, novel FLT3 inhibitors like crenolanib, which is potent, selective, and invulnerable to resistance-conferring kinase domain mutation, are developed [61]. In addition to FLT3-ITD mutation in nearly one third of AML patients [62], nowadays with the progress of more powerful FLT3 inhibitors being tested in many clinical trials, resistance-conferring point mutations like D835 and F691 have emerged during disease progression [63]. Crenolanib is a selective pan inhibitor active against both FLT3-ITD and FLT3-TKD D835 mutations, whereas most agents only have limited activity against the former. Crenolanib is a benzamidine quinolone derivative and currently a representative of the potent next-generation FLT3 TKIs.

#### Gilteritinib (ASP2215)

As potent as crenolanib, gilteritinib is also a selective next-generation FLT3 inhibitor with activity against both FLT3-ITD and FLT3-TKD mutations. A preclinical study compared gilteritinib with four other FLT3 inhibitors using immunoblotting and drew a conclusion that its in vitro efficacy is equal to or greater than the other TKIs (midostaurin, sorafenib, quizartinib, and crenolanib) and may be the most useful FLT3 inhibitor to date [64]. Worth mentioning, due to less activity against c-kit than quizartinib, gilteritinib has little myelosuppression.

#### Lestaurtinib

Lestaurtinib (CEP-701) is an orally bioavailable first-generation FLT3 inhibitor, as well as a potent inhibitor of JAK2 [65, 66]. Recently, a randomized assessment from UK AML 15 and AML 17 trials confirmed no statistically significant benefit observed in the combination of lestaurtinib with standard chemotherapy for newly diagnosed AML patients mostly younger than 60 years.

### NPM1 mutation

NPM1 mutations represent the most frequent genetic alteration in AML, which are found in approximately 25% of patients with de novo AML. It is associated with improved outcomes, and the mechanisms have not been clearly elucidated. NPM1 is a promising therapeutic target for AML, since NPM1 mutations represent founder genetic lesions in leukemogenesis. Some recent studies have shown conflicting results on the association between NPM1 mutation and the response to ATRA or arsenic trioxide (ATO) adjunct to standard chemotherapy [67–69]. Interestingly, it has also been suggested that ATRA and arsenic trioxide combination can selectively induce apoptosis and differentiation in NPM1-mutated cells, as well as promote leukemia regression in elderly patients unfit for induction chemotherapy [70, 71]. Furthermore, since NPM1-mutated leukemia cells are associated with increased CD33 expression [72], CD33 antibodies like gemtuzumab ozogamicin (GO) could be a targeted therapy for those NPM1-mutated patients with high CD33 expression. Finally, recent evidence has emerged that drugs such as dactinomycin, triggering a nucleolar stress response, may target NPM1-mutated AML [73].

### Epigenetic mutations and alterations

Lately, epigenetic alterations that are heritable and reversible in contrast to genetic changes represent a focus of interest with respect to therapeutic targets in AML. With a rapid advance in all kinds of sequencing, recurrent mutated genes involved in epigenetic regulation have been identified, including TET2, IDH1, IDH2, DNMT3A, and EZH2. Abnormal DNA methylation and histone modification are two main modes of epigenetic dysregulation.

#### DNMT inhibitors

DNA methylation is catalyzed by DNMTs. Recurrent mutations in DNMT3A are found in 6 to 36% of AML patients, which is hypothesized to act as dominant negatives in leukemogenesis [74]. HMAs inhibiting DNMTs are options for older patients who cannot tolerate intensive chemotherapy with lower toxicities and equal efficacy. As known, azacitidine (5-Aza) and decitabine are two HMAs currently approved for clinical use, both of which have shown clinical benefit in clinical trials [75–78]. A phase II study of older patients who were unfit for intensive chemotherapy treated with 10-day schedule of decitabine yielded a CR rate of 47%, without certain benefit observed in the combination of decitabine with HDAC valproic acid. Interestingly, patients harboring monosomy 7 or del(7q) had a higher response rate of 91% [79]. This study also proposed that higher pretreatment levels of miR-29b were associated with response (*P* = 0.02) to decitabine, allowing it to be a predictive marker and stratification tool in selection of older AML patients for this regimen. Further multicenter studied should be performed. Another single-institution trail suggested patients with unfavorable risk or TP53 mutations had significantly higher response rates to 10-day decitabine therapy despite their poor prognosis after cytotoxic chemotherapy [80]. In addition to this, the OS rate was similar among patients with unfavorable-risk and intermediate-risk cytogenetic profiles. It is worth mentioning that patients with TP53 mutations may not always be sensitive to single-agent decitabine treatment owing to the emergence of resistant subclones and incomplete mutation clearance. Still, decitabine should be considered as an important agent in the treatment of AML patients with TP53 mutations unfit for cytotoxic chemotherapy. To date, there are no therapies specifically targeting against DNMT3A.

Guadecitabine (SGI-110), as a second-generation HMA, is a dinucleotide of decitabine and deoxyguanosine resistant to cytidine deaminase and can prolong the exposure to decitabine in vivo. A phase I study assessed three treatment schedules of guadecitabine: daily schedule for 5 days continuously, weekly, and twice-weekly schedule. It was identified that the maximum demethylation was achieved with a dose of 60 mg/m^2^ per day for 5 days consecutively [81]. Likewise, a multicenter randomized phase I/II study accessing the safety and activity of two doses and schedules of guadecitabine in older AML patients also recommended the 60 mg/m^2^ guadecitabine in a 5-day regimen. A phase II study randomizing among 5-day regimen, 10-day regimen, and a combination of the 5-day schedule with idarubicin or cladribine is ongoing (NCT02096055), as well as a phase III study in progress to compare this 5-day schedule of guadecitabine with standard care. Also, another phase III randomized study of guadecitabine versus treatment choice in R/R AML has been initiated (NCT02920008). Anyway, therapeutic efficacy of guadecitabine will ultimately rely on a demonstrable improvement in OS; only then SGI-110 can be expected to become an alternative choice for patients ineligible for traditional induction chemotherapy due to old age, comorbidities, etc. [82].

Sapacitabine (CYC682) is a novel oral nucleoside analog. It is metabolized into the active metabolite CNDAC and incorporated into cellular DNA to exert anticancer activity by interfering with DNA synthesis and inducing cell cycle arrest. In a phase II trial, sapacitabine was administered to 60 AML patients aged 70 years or older from 12 centers in the USA, studying three dose schedules of sapacitabine: (A) 200 mg bid for 7 days, (B) 300 mg bid for 7 days, and (C) 400 mg bid on days 1–3 for 2 weeks. One-year OS was 35, 10, and 30% in three groups, respectively. The 30-day mortality was 13% and the 60-day mortality doubled [83]. In addition, SEAMLESS, a multicenter, randomized, phase III study of comparing sapacitabine alternating with decitabine to single agent decitabine in approximately 485 elderly patients aged 70 years or older, is ongoing (NCT01303796).

#### IDH inhibitors

IDH is one kind of enzyme that catalyzes the oxidative decarboxylation of isocitrate to alpha-ketoglutarate (α-KG), and the enzyme TET2 co-works with a-KG to convert 5-methylcytosine (5mC) to 5-hydroxymethylcytosine (5hmc), which promotes DNA demethylation. Mutant IDH (mIDH) enzymes convert α-KG to (R)-2-HG, which competitively inhibits a-KG-dependent enzymes including TET2. Besides, inactivating mutation in TET2 can lead to loss of function. Thus, both IDH and TET2 mutations can result in accumulation of 5mc and DNA hypermethylation and consequently promote AML.

Though prognostic impact of IDH gene mutations remains controversial, inhibitors targeted mIDH have been developed these days. Small-molecule mIDH inhibitors include AG-221, AG-120, AG-881, and IDH305, among which AG-221 and AG-120 have shown evidence of efficacy and are being tested in clinical trials. The first IDH2 inhibitor AG-221 is developed to inhibit mutant IDH2, reduce 2HG levels, and restore TET2 activity, thereby reversing 5mC accumulation in mouse mIDH AML models [84]. In ASH 2015, a phase I dose escalation and expansion study of AG-221 demonstrated an ORR of 41% and a true CR of 18% in patients with R/R AML [85]. AG-120 monotherapy was associated with an ORR of 35% in a similar study [86]. Additionally, methylation inhibitors like 5-Aza can inhibit the conversion of cytosine to 5mC in TET2-mutant AML in mice, thereby preventing 5mC from accumulation. In a study, a comparison was drawn between the effects of preventing DNA hypermethylation induced by genetic loss of TET2 and restoring TET2 activity by inhibiting mutant IDH2 in AML [87]. Both AG-221 and 5-Aza induced differentiation of leukemic cells, but neither significantly killed mutant cells. Therefore, targeting epigenetic dysregulation could be an effective therapeutic strategy in AML, while dual-pronged therapies such as combining epigenetic inhibitors with kinase-targeted therapies may be a better choice [88]. Nowadays, both AG-120 and AG-221 are being investigated in newly diagnosed AML patients with IDH mutations, in combination with induction and consolidation chemotherapy (NCT02632708) and azacitidine (NCT02677922).

#### HDAC inhibitors

Histone modifications include acetylation and methylation which are reversibly mediated by histone acetyltransferases, HDACs, HMTs, and histone demethylases, respectively. Histone acetylation increases the accessibility of transcription factors to gene regions and consequently promotes gene expression. Conversely, deacetylation leads to transcriptional repression and impaired hematopoietic differentiation, which can be inhibited by HDAC inhibitors (HDACIs). When used as a single agent in MDS and AML, HDACIs seem to be modest [89]. Considering the disappointing results of combined clinical trials recently [55, 90–93], it remains a challenge to find an optimal combination regimen of HDACIs with other agents. Other histone modifiers like BET inhibitors (OTX015), DOT1L inhibitors (EPZ-2676), and LSD1 inhibitors (GSK2879552) are being investigated as monotherapy in clinical trials and still need further exploration.

### Immunotherapy for AML

Cancer immunotherapy aims to stimulate the immune system to destroy tumors. Novel immunotherapies for AML mainly consist of monoclonal antibodies (mAbs), chimeric antigen receptor-engineered T cells (CAR T cells), and checkpoint inhibitors.

#### Monoclonal antibodies

Currently, the most encouraging therapeutic targets for AML are CD33 and CD123, which are both expressed in leukemic cells and normal hematopoietic cells. Due to off-tumor effects of aplasia and neutropenia, it is relatively more difficult to find an ideal target for AML than ALL. It was wildly known that GO, the first anti-CD33 mAb approved by the FDA in 2000, was withdrawn from market in 2010 due to early toxicity and little clinical benefit. Nevertheless, recent studies have demonstrated an improved survival in populations with favorable/intermediate-risk cytogenetics [94–97]. Since older AML patients are not suited to cytotoxic chemotherapy, best supportive care (BSC) including hydroxyurea or low-dose cytarabine is considered despite dismal outcomes [98]. A randomized phase III EORTC-GIMEMA AML-19 trial demonstrated a significant improved OS in older AML patients with single-agent low-dose GO (6 mg/m^2^ on day 1 and 3 mg/m^2^ on day 8), compared with BSC group (4.9 vs 3.6 months, *P* = 0.005). Subgroup analysis confirmed the prediction that GO would be most effective in patients with high CD33 expression [99]. Phase IV clinical trials for patients with relapsed AML are now ongoing (NCT02312037). Given the above, GO monotherapy could embody a new choice for elderly patients. Besides, the combination of azacitidine and GO in phase II studies also revealed encouraging remission and survival rates in elderly patients [100, 101].

At ASH 2015, another CD33 antibody known as SGN-33A, presented promising results in a phase I study in combination with hypomethylating drugs in older patients [102]. These encouraging results have promoted the phase III CASCADE study (NCT019002329) which attempts to evaluate SGN-33A combined with 5-Aza or decitabine for older adults with newly diagnosed AML. In comparison with GO, vadastuximab seems to have effective therapeutic results in poor-risk group in ongoing studies [103]. Other drugs like CSL362 and SL-401 that target CD123 are now being investigated in various studies and have shown some promising data [104, 105].

#### CAR T therapy

Closely linked with graft-versus-host disease (GvHD), graft-versus-leukemic (GvL) effect appears after HSCT, via which the donor T cell plays an important role in killing leukemia cells. Elderly patients are not suitable candidates for HSCT due to high toxicity and relapse rate [106], and the efficacy of HSCT could be enhanced by infusion of CAR T cells [107]. CARs targeting CD19 have demonstrated remarkable potency in B cell malignancies such as B-ALL. The success of CD19 CAR T lies in two factors: (1) massive expansion and persistence of infused CAR T cells with costimulators and (2) tolerability of CD19 B cell aplasia due to its limited expression on mature B cells [108]. As mentioned, it remains challenging to find an ideal AML target owing to its profound and intolerable hematopoietic toxicity. Most of the current antigens in AML treatment are just over-expression antigens, rather than true AML-specific surface antigens, which brings about fatal off-tumor toxicity [109]. Several studies have proved in mouse models that targeting with anti-CD123 CAR T-cells (CD123 CART) and anti-CD33 CAR T-cells (CD33 CART) had some anti-AML potency but severe myeloablation was inevitable [110–112]. Particularly, a preclinical study in a mouse model using CD123 CART showed that CD123 expressed more frequently than CD33, and CD123 CART mediated potent in vivo antileukemic effect as well as increased survival of the majority of animals [110]. Furthermore, it is worth mentioning that the persistence of CAR T cells is associated with both anti-tumor efficacy and prolonged myeloablation, which suggests that a following rescue HSCT strategy is imperative. Future investigations in CAR T therapy warrant more focus on selection of specific AML-related surface targets.

#### Checkpoint inhibitors

In normal situations, immune checkpoints act as protective mechanism against autoimmunity, while tumor cells take advantage of them to evade immune system response and mediate immune resistance [113]. Thus, checkpoint inhibitors work via unleashing suppressed immune responses [114]. Two key checkpoint receptors are programmed cell death protein 1 (PD1) and cytotoxic T-lymphocyte-associated antigen 4 (CTLA4), both of which have been used in preclinical AML models [115]. One group treated three relapsed AML patients after allo-HSCT with the PD-1 inhibitor nivolumab. Among these three patients, one achieved an ongoing CR, one experienced stabilization, and the third failed to respond. It suggested that targeting PD-1 might be an effective salvage therapy for relapsed AML after allo-HSCT, though the optimal dose of nivolumab to restore GvL effects without leading to severe GvHD still remains studying [116]. Phase II trials using single nivolumab or in combination with CTLA4 antibodies after allo-HSCT and chemotherapies are ongoing (NCT02532231, NCT02846376, NCT02464657).

## Conclusion

The fundamental goal of precision medicine is to integrate population-based molecular, clinical, and other data to make individual-based clinical decisions for patients [117]. It has been demonstrated that the new formulated chemotherapies, molecular targeted agents, and immunotherapies all have clinical activity as single agents, but the activity seems limited. Recent studies have confirmed that combining with chemotherapy or other new drugs may bring more benefit for AML patients. The treatment of AML remains a tough challenge despite advances in our understanding of molecular mechanism and prognostic impact. Therefore, many questions are still unsolved in the use of these new drugs, which indicates that both patient and disease status should be taken into consideration. Massive efforts are required to pave the way for precision medicine in the foreseeable future.

## Abbreviations

5hmc: 5-Hydroxymethylcytosine
5mC: 5-Methylcytosine
allo-HSCT: Allogeneic HSCT
AML: Acute myeloid leukemia
APL: Acute promyelocytic leukemia
ATO: Arsenic trioxide
ATRA: All-transretinoic acid
CAR T cells: Chimeric antigen receptor-engineered T cells
CR: Complete remission
CTLA4: Cytotoxic T-lymphocyte-associated antigen 4
DLT: Dose-limiting toxicity
DNMTs: DNA methyltransferases
EFS: Event-free survival
FLT3: Fms-like tyrosine kinase 3
GO: Gemtuzumab ozogamicin
GvHD: Graft-versus-host disease
GvL: Graft-versus-leukemic
HDAC: Histone deacetylase
HMAs: Hypomethylating agents
HSCT: Hematopoietic stem cell transplantation
ITD: Internal tandem duplications
LDAC: Low-dose cytarabine
mAbs: Monoclonal antibodies
mIDH: Mutant IDH
MTD: Maximum tolerated dose
OS: Overall survival
PD1: Programmed cell death protein 1
PDGFR: Platelet-derived growth factor receptor
PLKs: Polo-like kinases
R/R: Relapsed/refractory
TKD: Tyrosine kinase domain
TKIs: Tyrosine kinase inhibitors
VEGFR: Vascular endothelial growth factor receptor
WT: Wild type
α-KG: Alpha-ketoglutarate
